# Restricting lignin and enhancing sugar deposition in secondary cell walls enhances monomeric sugar release after low temperature ionic liquid pretreatment

**DOI:** 10.1186/s13068-015-0275-2

**Published:** 2015-07-04

**Authors:** Chessa Scullin, Alejandro G. Cruz, Yi-De Chuang, Blake A. Simmons, Dominique Loque, Seema Singh

**Affiliations:** Deconstruction Division, Joint BioEnergy Institute, Lawrence Berkeley National Laboratory, Berkeley, CA USA; Biological and Materials Science Center, Sandia National Laboratories, Livermore, CA USA; Advanced Light Source, Lawrence Berkeley National Lab, Berkeley, CA USA; Feedstocks Division, Joint BioEnergy Institute, Lawrence Berkeley National Laboratory, Berkeley, CA USA; Physical Biosciences Division, Lawrence Berkeley National Laboratory, Berkeley, CA 94720 USA; Joint BioEnergy Institute, 5885 Hollis Street, Emeryville, CA 94608 USA

**Keywords:** *Arabidopsis*, Biofuels, Cell wall, Lignin, Saccharification, Ionic liquid

## Abstract

**Background:**

Lignocellulosic biomass has the potential to be a major source of renewable sugar for biofuel production. Before enzymatic hydrolysis, biomass must first undergo a pretreatment step in order to be more susceptible to saccharification and generate high yields of fermentable sugars. Lignin, a complex, interlinked, phenolic polymer, associates with secondary cell wall polysaccharides, rendering them less accessible to enzymatic hydrolysis. Herein, we describe the analysis of engineered *Arabidopsis* lines where lignin biosynthesis was repressed in fiber tissues but retained in the vessels, and polysaccharide deposition was enhanced in fiber cells with little to no apparent negative impact on growth phenotype.

**Results:**

Engineered *Arabidopsis* plants were treated with the ionic liquid (IL) 1-ethyl-3-methylimidazolium acetate 1-ethyl-3-methylimidazolium acetate ([C_2_C_1_im][OAc]) at 10 % wt biomass loading at either 70 °C for 5 h or 140 °C for 3 h. After pretreatment at 140 °C and subsequent saccharification, the relative peak sugar recovery of ~26.7 g sugar per 100 g biomass was not statistically different for the wild type than the peak recovery of ~25.8 g sugar per 100 g biomass for the engineered plants (84 versus 86 % glucose from the starting biomass). Reducing the pretreatment temperature to 70 °C for 5 h resulted in a significant reduction in the peak sugar recovery obtained from the wild type to 16.2 g sugar per 100 g biomass, whereas the engineered lines with reduced lignin content exhibit a higher peak sugar recovery of 27.3 g sugar per 100 g biomass and 79 % glucose recoveries.

**Conclusions:**

The engineered *Arabidopsis* lines generate high sugar yields after pretreatment at 70 °C for 5 h and subsequent saccharification, while the wild type exhibits a reduced sugar yield relative to those obtained after pretreatment at 140 °C. Our results demonstrate that employing cell wall engineering efforts to decrease the recalcitrance of lignocellulosic biomass has the potential to drastically reduce the energy required for effective pretreatment.

**Electronic supplementary material:**

The online version of this article (doi:10.1186/s13068-015-0275-2) contains supplementary material, which is available to authorized users.

## Background

Liquid transportation biofuels derived from sustainable lignocellulosic biomass have the potential to significantly reduce greenhouse gas emissions relative to petroleum-derived fuels. While significant progress has been made in improving the economic viability and commercial scalability of renewable biofuels, there remain significant challenges that must be addressed before these processes reach their full potential [1–3]. These challenges include the relatively low energy density of the biomass feedstocks, the recalcitrance of the plant cell walls to enzymatic hydrolysis [1–3], and the current high cost of pretreatment required to reduce this recalcitrance [4]. Biomass pretreatments that use certain ionic liquids (ILs), such as 1-ethyl-3-methylimidazolium acetate ([C_2_C_1_im][OAc]), have been shown to help overcome biomass recalcitrance by increasing surface area and by partially or completely solubilizing the cell wall, decreasing cellulose crystallinity, increasing cellulose accessibility, and/or removing lignin [4–10]. One technique to monitor IL pretreatment is imaging the autofluorescence of biomass during IL pretreatment. These imaging studies have shown that a key step in biomass pretreatment using [C_2_C_1_im][OAc] is cell wall swelling [9, 11]. The composition of the biomass and extent of delignification further affect biomass recalcitrance and saccharification kinetics [4, 6, 12–16]. Increasing the accumulation of polysaccharides in biomass and improving biomass digestibility would have significant beneficial impacts on the cost of lignocellulosic biofuel production, both by increasing fermentable sugar yield per acre and reducing the severity of pretreatment [2, 17].

The secondary cell walls in Arabidopsis are composed of cellulose (40 %), matrix polysaccharides (~35 %) and lignin (~20 %; primarily G and S units) [18–20]. Secondary cell walls are deposited on top of the primary cell wall in specific tissues (e.g., vessels and fibers) to provide rigidity and strength. Recently, a new approach using synthetic biology was developed in *Arabidopsis* to decrease lignin content in fibers while retaining its deposition in vessels [21, 22]. In contrast to most approaches used to reduce lignin content [23–25], this one had no obvious impact on phenotype and plant growth. The engineering consisted of replacing the promoter controlling the expression of the second gene in the lignin pathway (*C4H*) that controls the metabolic flux of lignin biosynthesis via the vessel-specific promoter corresponding to the transcription factor *VND6*. This low lignin line was further engineered to enhance polysaccharide deposition in plant fiber cells using an artificial positive feedback loop technology that allows for the targeted overexpression of a key transcription factor, NST1, known to control secondary cell wall deposition in fibers [21, 26]. The combination of both approaches resulted in decreased biomass recalcitrance that generated higher yields of fermentable sugars on a per plant basis after hot water pretreatment followed by enzymatic hydrolysis [21].

To understand the full impact of these cell wall modifications on IL pretreatment, we investigated [C_2_C_1_im][OAc] biomass pretreatment on one low lignin line (*LLL*, line #135 in [21]) and two low lignin high polysaccharide lines (*LLHPL1,* line #89 in [21] and *LLHPL2*, line #60 in [21]). *LLHPL1* and *LLHPL2* were selected due to their different levels of polysaccharide accumulation [21]. The main objectives were to gain insight of the effect of cell wall modification on biomass deconstruction using ILs and to determine if the IL pretreatment process could be carried out at lower temperatures as a result of these modifications. We report the impact of these engineered lines relative to wild type (*WT*) in terms of pretreatment efficacy, sugar yields, and mass balances for IL pretreatment at 70 and 140 °C using [C_2_C_1_im][OAc] followed by saccharification using commercially available enzyme mixtures.

## Results and discussion

**Fig. 1:**
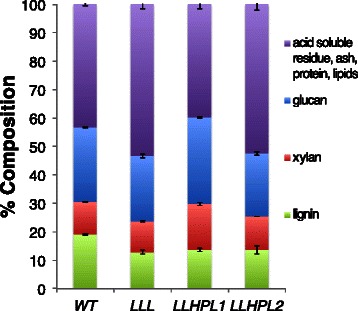
Compositional profile of the four *Arabidopsis* engineered lines (*WT*, *LLL*, *LLHPL1*, *LLHPL2*)

Mature, senesced stems (corresponding to the main stems and side branches depleted of seeds and cauline leaves) from multiple plants of the *WT*, *LLL*, *LLHPL1*, and *LLHPL2* Arabidopsis lines grown under the same conditions were collected and milled, and the chemical composition was quantified. As previously reported, all the lines (*LLL*, *LLHPL1*, and *LLHPL2*) harboring the *pVND6::C4H* construct, exhibit a significantly lower lignin content (12.9 to 14 %) compared to that of *WT* (19.1 %) and had no visible phenotypic differences (Table 1, Fig. 1) [21]. As expected, *LLHPL1* shows an increase in the amount of both glucose 30.4 % and xylose 16.1 % present versus *WT* (26.1 and 11.4 % respectively). The *LLHPL2* showed only a minor increase in xylose, 11.7 %, for the bulk composition and a significant decrease in the amount of glucose present, 22.1 %, where previously it was found to have a significant increase on a per plant scale [21]. Both the *LLL* and *LLHPL2* engineered *Arabidopsis* lines exhibit a significant increase in acid soluble residue (ASR), while *LLHPL1* had an increase in glucose with little change in ASR compared to *WT* (Table 1).

**Table 1:** Initial compositional analysis for each *Arabidopsis* engineered line studied

Untreated composition				
	% Glucose	% Xylose	% Lignin	% ASR
*WT*	26.1 ± 0.1	11.4 ± 0.1	19.1 ± 0.3	43.4 ± 0.5
*LLL*	23.0 ± 0.7^**^	10.8 ± 0.2	12.9 ± 0.8^**^	53 ± 2^**^
*LLHPL1*	30.4 ± 0.4^**^	16.1 ± 0.5^**^	13.7 ± 0.6^**^	40 ± 2
*LLHPL2*	22.1 ± 0.5^**^	11.7 ± 0.1	14 ± 2^**^	53 ± 2^**^

There was an overall significant difference in the concentration of glucose, xylose, lignin, and ASR (acid soluble residue, ash, protein) F_(3,12)_ = 150.87, *P* < 0.0001, F_(3,12)_ = 340.36, *P* < 0.0001, F_(3,12)_ = 28.65, *P* < 0.0002, F_(3,12)_ = 100.54, *P* < 0.0001. ANOVA with a Tukey’s HSD post-hoc test was used to determine overall statistics, and results of the comparison to *WT* from the Tukey’s HSD post-hoc test are shown in the table. Values expressed ± SD

^**^ *P* < 0.01

**Fig. 2:**
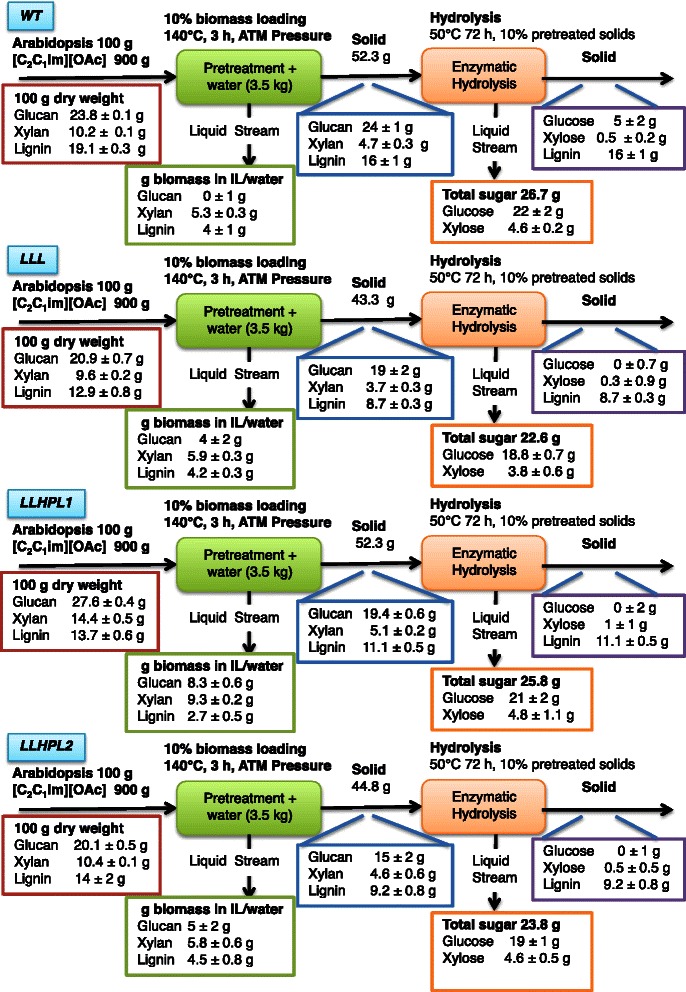
Mass balance of [C_2_C_1_im][OAc] pretreatment of the four *Arabidopsis* lines (*WT*, *LLL*, *LLHPL1*, and LLHPL2) at 140 °C for 3 h. Mass balance adjusted to 100 g starting biomass. Values presented as ±SD

We pretreated the *WT* and the engineered strains with [C_2_C_1_im][OAc] at 10 % (w/w) biomass loading at 140 °C for 3 h (Fig. 2) [8, 10, 27, 28]. The pretreated slurry was washed with water as an anti-solvent, precipitating a solid. The lignin concentrations of the pretreated solids from the reduced lignin lines were confirmed to be significantly lower than *WT* (~20 % lignin in the engineered lines and ~30 % lignin in the *WT*, Table 2) with insignificant differences in the amount of glucose and xylose removed for the engineered lines (Table 2). The *WT* had a significantly higher glucan recovery in the after IL pretreatment, as compared to the engineered lines where glucan recoveries of 86, 70, and 74 % were quantified for *LLL*, *LLHPL1*, and *LLHPL2*, respectively. Less than 50 % of xylan was recovered in the solids after pretreatment for all of the *Arabidopsis* lines tested (Table 2), and all three of the reduced lignin lines had a significant increase in ASR in the recovered biomass after IL pretreatment as compared to the *WT* (Table 2).

**Table 2:** Percent recovered solid composition after pretreatment at 140 °C for 3 h with [C_2_C_1_im][OAc] at 10 % (w/w) biomass loading as a percent of starting biomass

	Solids recovery		140 °C 3 h	
	% Glucose	% Xylose	% Lignin	% ASR
*WT*	101 ± 6	47 ± 3	82 ± 5	17 ± 2
*LLL*	86 ± 7	39 ± 3	68 ± 6	23 ± 1^*^
*LLHPL1*	70 ± 3^**^	34 ± 1^*^	81 ± 14	38 ± 5^**^
*LLHPL2*	74 ± 10^**^	44 ± 6	70 ± 13	28 ± 3^**^
Pretreated solids composition 140 °C for 3 h				
	% Total solids	% Glucose	% Xylose	% Lignin
*WT*	52 ± 3	51 ± 1	10 ± 2	30 ± 2
*LLL*	43 ± 4	46 ± 2	10 ± 2	20 ± 2^*^
*LLHPL1*	52 ± 2	41 ± 6	11 ± 1	21 ± 4^**^
*LLHPL2*	44 ± 6	37 ± 3	12 ± 3	21 ± 3^**^

All values presented as ±SD. There was an overall significant difference in % recovery of glucose, xylose, and ASR (acid soluble residue, ash, and protein) in the recovered solids, F_(3,12)_ = 12.86, *P* < 0.002, F_(3,12)_ = 7.37, *P* < 0.01 and F_(3,12)_ = 32.87, *P* < 0.0001. There was a non-significant difference in the lignin recovery in the solids, F_(3,12)_ = 1.03, *P* = 0.43. Composition of recovered solids after pretreatment with [C2C1im][OAc] for 140 °C 3 h at 10 % (w/w) biomass loading. Glucose, xylose, and lignin reported as a percent of recovered biomass ±SD. There was an overall significant difference in % total solids and lignin, F_(3,12)_ = 5.08, *P* < 0.05, F_(3,12)_ = 9.74, *P* < 0.005. There was no overall significance for the % composition glucose or xylose F_(3,12)_ = 6.22, *P* = 0.05, F_(3,12)_ = 0.35, *P* = 0.79. ANOVA with a Tukey’s HSD post-hoc test was used to determine overall statistics, and results of the comparison to *WT* from the post-hoc test are shown in the table

^*^ *P* < 0.05; ^**^ *P* < 0.01

The recovered solids from the *Arabidopsis* lines after [C_2_C_1_im][OAc] pretreatment were then saccharified using a commercial cellulase (CTec2) and hemicellulase (HTec2) enzyme mixture [10]. The yields of glucan after saccharification for *LLL*, *LLHPL1*, and *LLHPL2* were >95 % and significantly higher than those obtained from samples with no pretreatment (Table 3). There was no difference in the saccharification efficiency for xylan yields between the three modified plant lines. This resulted in final glucose yields of 69 to 87 % recovery in terms of the initial amount present in the samples before pretreatment. These glucose yields were not significantly different between the *WT*, *LLL*, and *LLHPL2* samples but were significantly lower for glucose and xylose released from *LLHPL1* compared to the *WT,* as well as xylose released from the *LLL* sample (Table 3, Fig. 2).

**Table 3:** Enzymatic saccharification efficiency of Arabidopsis engineered line versus pretreatment condition

Enzymatic saccharification 10 % loading for 72 h					
		% Glucose	% Xylose	% Glucose recovery	% Xylose recovery
*WT*	Untreated	31 ± 3	17 ± 3	31 ± 3	17 ± 3
	10 %, 70 °C, 5 h	67 ± 20	1.0 ± 0.3	62 ± 11	1.0 ± 0.2
	10 %, 140 °C, 3 h	84 ± 6	87 ± 2	84 ± 1	41 ± 2
*LLL*	Untreated	46 ± 4^**^	33 ± 1^**^	46 ± 4^**^	33 ± 1^**^
	10 %, 70 °C, 5 h	76 ± 7	46 ± 5^**^	76 ± 5	46 ± 4^**^
	10 %, 140 °C, 3 h	95 ± 4	92 ± 7	82 ± 4	35 ± 4^**^
*LLHPL1*	Untreated	48.4 ± 0.7^**^	30.2 ± 0.2^**^	48 ± 0.4^**^	30 ± 0.3^**^
	10 %, 70 °C, 5 h	79 ± 4^**^	58 ± 3^**^	63 ± 4	48 ± 2^**^
	10 %, 140 °C, 3 h	99 ± 7^*^	83 ± 9	69 ± 3^*^	30 ± 3
*LLHPL2*	Untreated	53 ± 3^**^	31 ± 1^**^	53 ± 3^**^	31 ± 1^**^
	10 %, 70 °C, 5 h	81 ± 4^**^	55 ± 5^**^	79 ± 1^*^	58 ± 1^**^
	10 %, 140 °C, 3 h	117 ± 6^**^	89 ± 6	87 ± 8	39 ± 2

Enzymatic saccharification efficiency reported as percent of theoretical in the saccharification (released as percent from pretreated biomass) and final recovery % from concentration in initial solids (sugar recovery * enzymatic efficiency), from the cellulose and hemicellulose mixtures CTec2 and HTec2 (20 mg/g and 2 mg/g loading for 72 h). All values presented as ±SD. There was an overall significant difference of the % glucose and xylose released from untreated biomass during enzymatic saccharification between the *WT* and the three engineered lines, F_(3,12)_ = 30.59, *P* < 0.0001, F_(3,12)_ = 66.83, *P* < 0.0001, xylose for the 70 °C pretreated biomass, F_(3,12)_ = 139.36, P < 0.0001, and glucose for the 140 °C pretreated biomass, F_(3,12)_ = 18.57, *P* < 0.001. There was a non-significant differences for the % saccharification efficiency for glucose for the 70 °C pretreatment F_(3,12)_ = 0.95, *P* = 0.46 and for xylose for the 140 °C pretreatment F_(3,12)_ = 0.85, *P* = 0.50. There were significant differences both the glucose and xylose recoveries at each pretreatment condition, untreated (F_(3,12)_ = 30.6, *P* < 0.0001, F_(3,12)_ = 66.8, *P* < 0.0001), 70 °C (F_(3,12)_ = 4.35, *P* < 0.05 F_(3,12)_ = 355.29, *P* < 0.0001) and 140 °C (F_(3,12)_ = 7.93, *P* < 0.01, F_(3,12)_ = 9.38, *P* < 0.01). ANOVA with a Tukey’s HSD post-hoc test was used to determine overall statistics, and results of the comparison to *WT* from the post-hoc test are shown in the table

^*^ *P* < 0.05; ^**^ *P* < 0.01

**Fig. 3:**
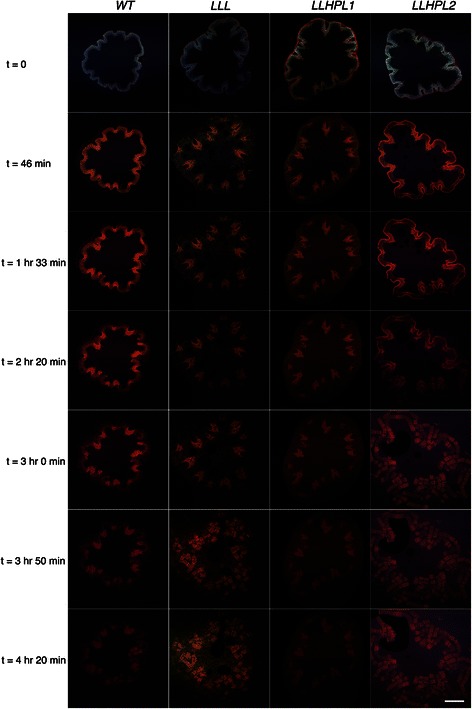
Confocal fluorescence imaging of *Arabidopsis* during [C_2_C_1_im][OAc] pretreatment at 140 °C. Autofluorescence of 100 μm slices of the stems from four Arabidopsis lines during [C_2_C_1_im][OAc] pretreatment at 140 °C over 4.3 h. Horizontal panels show the different *Arabidopsis* lines. Vertical panels show the progression of the time course of [C_2_C_1_im][OAc] pretreatment on *Arabidopsis* with a temperature ramp from ambient conditions to 140 ± 5 °C occurring during time 0 to 46 min, scale bar 500 μm

**Fig. 4:**
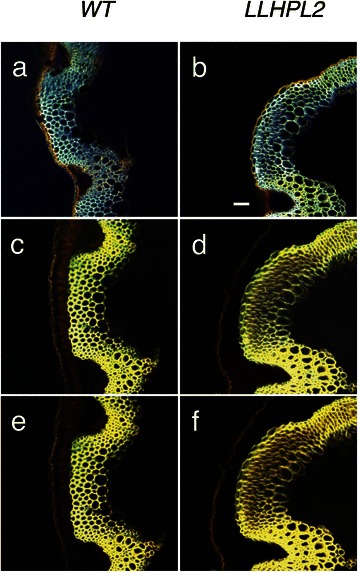
Confocal fluorescence imaging of *Arabidopsis* during [C_2_C_1_im][OAc] pretreatment at 70 °C for 11 h. Heating occurred during ramp from room temperature to 70 °C during the first 30 min of imaging. Horizontal panels show comparison of *WT* versus the engineered line *LLHPL2* while the vertical panels show selected images of the time course (**a**, **b**) 0, (**c**, **d**) 5 h, (**e**, **f**) 10 h, scale bar 50 μm

All of the *Arabidopsis* samples were observed to swell during IL pretreatment at 140 °C for 3 h (see Additional files 1, 2, 3, and 4: Movies 1–4). The observed rate of dissolution due to [C_2_C_1_im][OAc] pretreatment, however, was slower for the *WT* than the engineered lines (Fig. 3, Additional file 5: Figure S1). Due to the relatively minor differences observed in the rate and extent of dissolution at 140 °C, the temperature was reduced to 70 °C to determine if there were any significant differences observed in swelling and dissolution between the *WT* and *LLHPL2*. At this set of pretreatment conditions, there was an initial swelling step observed after 1 h of pretreatment, followed by the onset of extensive swelling after 3–4.5 h (Additional file 6: Figure S2, Additional files 7 and 8: Movie 5 and 6). Based on these results, a pretreatment incubation of 5 h at 70 °C was selected as the new pretreatment condition (Fig. 4).

**Fig. 5:**
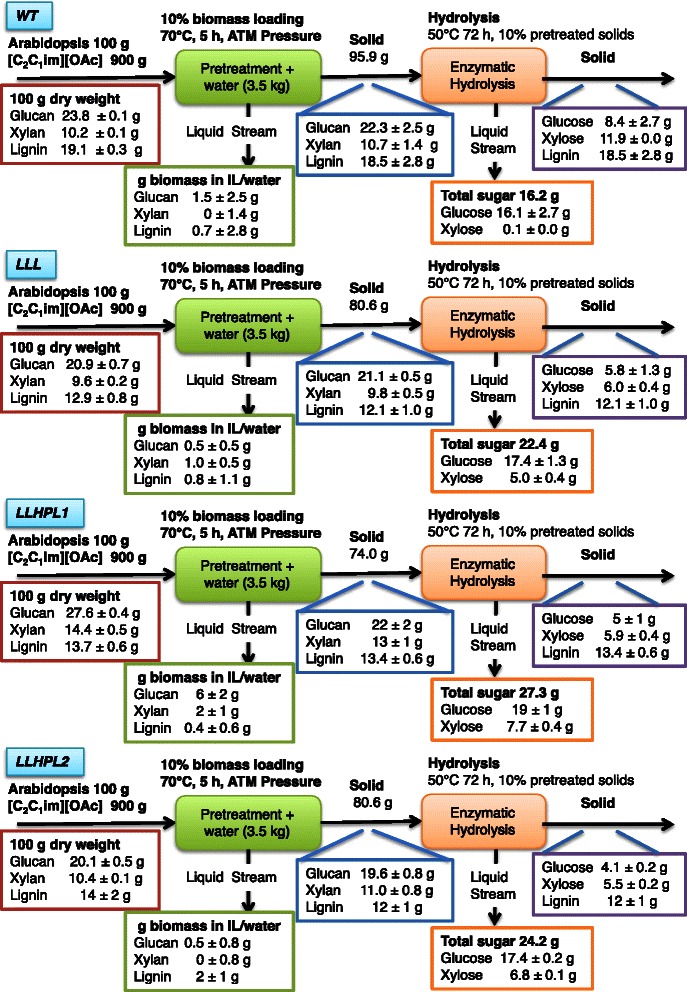
Mass balance of [C_2_C_1_im][OAc] pretreatment of the four Arabidopsis lines (*WT*, *LLL*, *LLHPL1*, and LLHPL2) at 70 °C for 5 h. Mass balanced adjusted to 100 g starting biomass. Values presented ±SD

The *Arabidopsis* lines *WT*, *LLL*, *LLHPL1*, and *LLHPL2* were pretreated in [C_2_C_1_im][OAc] at 70 °C for 5 h. The pretreated plant biomass was then precipitated and analyzed for composition (Fig. 5, Table 4). All of the lines had significantly lower solid recoveries (70.7 to 80.6 %) than those of the *WT* (96 %, Table 4), yet the three engineered lines had similar glucose and xylose recoveries in the pretreated solids as the *WT* (*WT* >94 % glucose, >106 % xylose, relative to initial biomass, Table 4). Furthermore, all of the *Arabidopsis* lines had minimal lignin removal (between 3 to 11 %) after pretreatment (Table 4).

**Table 4:** Percent recovered solid composition after pretreatment at 70 °C for 5 h with [C_2_C_1_im][OAc] at 10 % (w/w) biomass loading as a percent of starting biomass

Pretreated biomass solids recovery 70 °C 5 h				
	% Glucose	% Xylose	% Lignin	% ASR
*WT*	94 ± 10	106 ± 14	97 ± 15	94 ± 20
*LLL*	101 ± 2	102 ± 5	94 ± 8	66 ± 10
*LLHPL1*	80 ± 10	84 ± 8	97 ± 5	59 ± 20
*LLHPL2*	98 ± 4	106 ± 8	89 ± 7	50 ± 3^*^
Pretreated biomass composition 70 °C 5 h				
	% Total solids	% Glucose	% Xylose	% Lignin
*WT*	96 ± 10	26 ± 4	13 ± 2	19 ± 3
*LLL*	80.6 ± 0.9^**^	31 ± 1	13.7 ± 0.7	15 ± 2
*LLHPL1*	74 ± 1^*^	33 ± 4	19 ± 2^*^	18.1 ± 0.9
*LLHPL2*	70.7 ± 0.7^*^	29 ± 2	17 ± 1^*^	17 ± 1

Values presented as ±SD. There was an overall significant difference in % recovery of glucose and ASR (acid soluble residue, ash, and protein) in the recovered solids, F_(3,12)_ = 5.01, *P* < 0.03 and F_(3,12)_ = 4.07, *P* < 0.05. There was a non-significant difference in the % xylose and lignin recovery in the solids, F_(3,12)_ = 3.89, *P* = 0.06 and F_(3,12)_ = 0.44, *P* = 0.73. Composition of recovered solids after 70 °C 5 h [C2C1im][OAc] pretreatment. Glucose, xylose, and lignin were reported as the relative composition of recovered biomass. Pretreatment was done at 10 % (w/w) biomass. There was an overall significant difference in % total solids and xylose, F_(3,12)_ = 11.52, *P* < 0.005, F_(3,12)_ = 8.48, *P* < 0.01. There was no overall significance for the % composition glucose or lignin F_(3,12)_ =2.57, *P* = 0.13 and F_(3,12)_ = 3.1, *P* = 0.09. ANOVA with a Tukey’s HSD post-hoc test was used to determine overall statistics, and results of the comparison to *WT* from the post-hoc test are shown in the table

^*^ *P* < 0.05; ^**^ *P* < 0.01

**Fig. 6:**
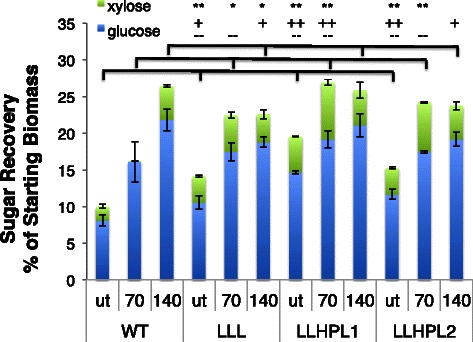
Comparison of glucose and xylose recovery after enzymatic saccharification as a percent of original biomass for [C_2_C_1_im][OAc] pretreatment. Glucose and xylose recovery after 70 °C for 5 h and 140 °C for 3 h compared to the untreated (ut) for all of the *Arabidopsis* lines. There was an significant difference in total sugar released per starting biomass between the *Arabidopsis* lines at each pretreatment temperature, untreated (F_(3,12)_ = 72.44, *P* < 0.0001), 70 °C (F_(3,12)_ = 19.45, *P* < 0.0005), and 140 °C (F_(3,12)_ = 5.86, *P* < 0.05). This was in part due to significant differences between groups in glucose recovery per starting biomass for all three pretreatment conditions untreated (F_(3,12)_ = 47.2, *P* < 0.0001), 70 °C (F_(3,12)_ = 7.86, *P* < 0.01), and 140 °C (F_(3,12)_ = 6.62, *P* < 0.01). There was also significant difference in xylose recovery per starting biomass between the lines for two of the three pretreatment conditions untreated (F_(3,12)_ = 134.12, *P* < 0.0001) and 70 °C (F_(3,12)_ = 404.71, *P* < 0.0001). There was not a significant difference in xylose release per starting biomass at 140 °C (F_(3,12)_ = 3.43, *P* = 0.07). ANOVA with a Tukey’s HSD post-hoc test and the Tukey’s HSD post-hoc test are shown in the figure for the comparison to *WT* (total sugar, *P* < 0.05, *; *P* < 0.01, **; glucose, *P* < 0.05, +; *P* < 0.01, ++; xylose, *P* < 0.05, −; *P* < 0.01, --), additional post-hoc test comparisons reported in Additional file 9: Table S1 and S2

The recovered solids from the different *Arabidopsis* lines after [C_2_C_1_im][OAc] pretreatment at 70 °C for 5 h were then saccharified. While there was less than 11 % removal of lignin, glucose yields of 76, 79, and 81 % were obtained for *LLL*, *LLHPL1*, and *LLHPL2*, respectively, and the saccharification efficiency was significantly greater for *LLHPL1* and *LLHPL2* than that of *WT* (67 %, Table 3). The resulting release of glucose relative to initial levels in the biomass was 62 % of the initial glucose for the *WT*, 76 % for the *LLL*, 63 % for the *LLHPL1*, and 79 % for the *LLHPL2* (Table 3, Fig. 5). There was minimal detectable xylose released (1 %) during saccharification for the *WT*; however, the three engineered lines had a significantly higher xylose yields of 46 to 58 %. In addition to the high recovery of glucose (63–79 %) and xylose (46–58 %) at the lower pretreatment temperature, the enhanced concentration of cellulose and hemicellulose per gram of starting biomass resulted in higher monomeric sugar release in all of the engineered lines (Figs. 2, 5, and 6). Both *LLHPL1* and *LLHPL2* have significantly increased total sugar recovery (27.3 and 24.2 g total sugar per 100 g starting biomass) as compared to the 16.2 g total sugar per 100 g starting biomass of the *WT* (Fig. 6, Additional file 9: Tables S1 and S2).

While there are similar recoveries and enhanced total sugar release, the saccharification kinetics are slower for the biomass pretreated at 70 °C than those pretreated at 140 °C (Table 5). After pretreatment at 70 °C for 5 h, the initial rate of glucose release for the *WT* was 86 mg/L/min, and the rates for the three engineered lines were between 40 to 52 mg/L/min. The rate of xylose release was below the detectable limit for *WT*, while the initial rate of release for xylose was significantly higher, between 46 to 68 mg/L/min, for the engineered lines. As the composition of both LLHPL1 and LLHPL2 are different, so were the rates of sugar released during saccharification. [C_2_C_1_im][OAc] pretreatment at 70 °C for 3 days has been shown previously to release less sugar than pretreatment at 140 °C for 3 h [29]. The reduced lignin *Arabidopsis* lines, however, all show increased sugar release after pretreatment at 70 °C, highlighting the impact of plant cell wall modifications on pretreatment severity and related energy requirements.

**Table 5:** Rate of enzymatic saccharification as calculated by release during the first 30 min of enzymatic hydrolysis with both the cellulase and hemicellulase mixtures CTec2 and HTec2

	Rate of enzymatic saccharification 10 % loading at 72 h		
		Rate glucose	Rate xylose
		(mg/L/min)	(mg/L/min)
*WT*	Untreated	43 ± 2	51 ± 10
	10 % 70 °C, 5 h	86 ± 16	n.d.
	10 % 140 °C, 3 h	196 ± 7	96 ± 42
*LLL*	Untreated	30 ± 0.5	41 ± 7
	10 % 70 °C, 5 h	41 ± 7^*^	46 ± 1^**^
	10 % 140 °C, 3 h	255 ± 10^*^	154 ± 33
*LLHPL1*	Untreated	54 ± 7	58 ± 20
	10 % 70 °C, 5 h	52 ± 8^*^	68 ± 9^**^
	10 % 140 °C, 3 h	271 ± 13^*^	221 ± 16^*^
*LLHPL2*	Untreated	19 ± 10	41 ± 20
	10 % 70 °C, 5 h	40 ± 16^**^	62 ± 13^**^
	10 % 140 °C, 3 h	221 ± 16	146 ± 50

Values presented ±SD. There were significant differences between *Arabidopsis* lines for both the initial glucose and xylose rates for solids pretreated at 70 °C (glucose, F_(3,12)_ = 8.8, *P* < 0.01 and xylose, F_(3,12)_ = 43.6, *P* < 0.0001), and solids pretreated at 140 °C (glucose, F_(3,12)_ = 7.35, *P* < 0.05 and xylose, F_(3,12)_ = 5.66, *P* < 0.05). There was no significant difference of initial rate of xylose release between the groups in untreated (xylose, F_(3,12)_ = 0.62, *P* = 0.62), but there was a significant difference between groups for initial rate of glucose release (glucose, F_(3,12)_ = 11.22, *P* < 0.05). ANOVA with a Tukey’s HSD post-hoc test was used to determine overall statistics, and results of the comparison to *WT* from the post-hoc test are shown in the table

*n.d.* not detectable

^*^ *P* < 0.05; ^**^ *P* < 0.01

## Conclusion

The impact of engineering secondary cell wall structure in *Arabidopsis* with a selective reduction of lignin and an enhancement of cellulose accumulation was evaluated in terms of pretreatment efficacy, sugar yields, and energy requirements. The reduced lignin *Arabidopsis* engineered lines resulted in high levels of monomeric sugar release at lower pretreatment temperatures as compared to the wild type. Ionic liquid pretreatment of the engineered *Arabidopsis* using [C_2_C_1_im][OAc] at 70 °C for 5 h resulted in improved saccharification efficiency and increased hemicellulose recovery for the pretreated biomass and produced similar total sugar yields as compared to those obtained after pretreatment at 140 °C for 3 h. The similar sugar recovery obtained for the engineered lines at the lower temperature pretreatment supports the hypothesis that reducing lignin can reduce the necessary severity of pretreatment needed and increased polysaccharide deposition can increase glucose recovery on a mass basis.

Secondary cell wall regulatory networks are only partially understood and seem to be conserved across many species from dicot to monocot plants [30–32]. For example, an *Arabidopsis nst1/nst3* double T-DNA insertional mutant lacking expression of both NST1 and NST3 transcription factors that control secondary cell wall deposition in fiber cells could be complemented by the expression of NST1 transcription factor orthologs derived from poplar or rice under the control of the *Arabidopsis* NST1 promoter [33, 34]. This had an effect on the ASR amounts between the engineered lines, which could be important for pretreatment and sugar recovery. This suggests that a similar approach for cell wall engineering could be implemented into other vascular plant species to enhance polysaccharide deposition in secondary cell walls. The different levels of sugar recovery between *LLHPL1* and *LLHPL2* demand further investigations into the optimal expression levels and patterns of C4H and NST1. Using this selective strategy to reduce lignin deposition and enhance carbohydrate composition of specific cellular structures in a more diverse group of vascular plants could create higher yielding feedstocks that require less energy to process, thereby, improving the overall economics of biofuel production.

## Methods

### Plant biomass

Wild type *Arabidopsis thaliana* (ecotype Columbia) and the three engineered lines named *LLL*, *LLHPL1*, and *LLHPL2* correspond to *c4h* + *pVND6::C4H*, *c4h* + *pVND6::C4H-pIRX8::NST1* line # 89 and line # 60, respectively, in Yang et al. [21]. The wild type *Arabidopsis* ecotype Col0 (*WT*) is our reference plant. The *pVND6::C4H* gene construct was used to complement the *Arabidopsis c4h* lignin mutant (*ref3-2*) [35, 36] and correspond to replacing the promoter for the second gene (C4H) in the lignin synthesis pathway with a promoter that is primarily expressed in vessel cells. This *LLL* plant line (*c4h* lignin mutant harboring the *pVND6::C4H* gene construct) was further engineered with pIRX8::NST1 construct [21] corresponding to the artificial positive feedback loop to increase secondary cell wall polysaccharide deposition. Two independent lines were generated [21] and were named *LLHPL1* and *LLHPL2* in this study. The lines *LLHPL1* and *LLHPL2* have been previously characterized, while having the same constructs, they have unique pIRX8::NST1 construct insertion sites resulting in compositional differences on a per plant basis [21].

*Arabidopsis* plants were grown in soil under short-day conditions for 5 weeks (10 h:14 h/light:dark cycle) before being transferred to long-day growth conditions (14 h:10 h/light:dark cycle) until mature at 150 μmol/m^2^/s, 22 °C, and 60 % humidity. The *Arabidopsis* main stems and side branches depleted of seeds and cauline leaves were pooled and milled to 40 mesh (0.255–0.451 mm) by a Wiley mill. All experiments were done in triplicate from different samples of the milled biomass.

### IL pretreatment

1-ethyl-3-methylimidazolium acetate, [C_2_C_1_im][OAc], was purchased from BASF (lot no. 08–0010, purity >95 %, BasionicsTM BC-01, BASF, Florham Park, NJ, USA) and used as the IL for all pretreatments. The *Arabidopsis* was stored at 4 °C in a cold room before use. *Arabidopsis* was pretreated with [C_2_C_1_im][OAc] at both 70 °C for 5 h and 140 °C for 3 h using a previously published protocol [8, 10]. Biomass loading in [C_2_C_1_im][OAc] was 10 % (w/w) with 2 g of starting biomass for each replicate.

After pretreatment, the samples were thoroughly mixed, and hot water as an anti-solvent was added at 3.5 times the initial total mass (of both biomass and IL) to recover any solubilized biomass. The mixture of IL, water, and biomass was centrifuged to separate the solid (biomass) and liquid ([C_2_C_1_im][OAc] and water) phases. The recovered solid was lyophilized (Labconco FreeZone^(12)^, Kansas City, MO, USA) and used for analysis.

### Compositional analysis

#### Total sugar analysis

Structural carbohydrates (including glucan and xylan) of *Arabidopsis*, before and after pretreatment (Tables 1, 2, and 4), were determined according to the two-step acid hydrolysis procedure of the National Renewable Energy Laboratory (NREL) [37]. Carbohydrates were diluted 100 fold and analyzed by HPLC. All values are reported ± one standard deviation (SD) unless noted.

### Lignin analysis

Acid insoluble lignin content of the untreated and pretreated *Arabidopsis* samples was determined using the two-step acid hydrolysis procedure of the National Renewable Energy Laboratory (NREL) [37]. All values are given with SD unless noted.

### Enzymatic saccharification

Enzymatic saccharification of pretreated and untreated *Arabidopsis* samples was carried out at 50 °C and 150 rpm in a reciprocating shaker (Enviro-Genie, Scientific Industries). Hydrolysis reactions were carried out in 5 mL of 50 mM sodium citrate buffer (pH of 4.8) with 10 % biomass loading. The glucan content in the solution was maintained at 5 g glucan per liter. For hydrolysis reactions, 20 mg protein/g glucan of Cellic® CTec2 (Novozymes, Davis, CA, USA) and 2 mg protein/g xylan of Cellic® HTec2 (Novozymes) were used. To monitor hydrolysis kinetics, 60 μL of the supernatant was taken at specific time intervals (0, 0.5, 1, 3, 6, 24, 48, and 72 h). The supernatants were centrifuged at 10,000 g for 5 min, and the released sugars in the supernatant were measured using solutions of D-glucose as calibration standards and high performance liquid chromatography. The untreated *Arabidopsis* controls were run concurrently with the 140 °C samples to eliminate potential variances in temperature, humidity, or mixing. The initial rate of hydrolysis was calculated based on the sugar released in the first 30 min of hydrolysis [10]. The supernatant collected after 72 h of hydrolysis was analyzed with HPLC for the enzymatic efficiency. All assays were performed with three replicates.

### Confocal fluorescence imaging

*Arabidopsis* samples from random sections of stem plant with similar diameter were sliced at 100 μm with a vibratome (Leica VT1000S, Microsystems Inc. Buffalo Grove, IL, USA). These sections were then stored at 4 °C until used in the imaging study. Slices were placed between a coverslip and slide with enough [C_2_C_1_im][OAc] to wet each sample (about 150 μL) and a thermocouple. The slide was placed in a temperature controlled (LakeShore model 331, Westerville, OH, USA) in-house heater (Advanced Light Source, LBNL). Samples were started at room temperature and ramped to the specified temperature during imaging to using the high heat setting. Samples, on average, reached the specified temperature (70 or 140 °C) before 30 min and fluctuated ± 5 °C. Autofluorescent images during heating were collected with a Zeiss LSM 710 confocal system mounted on a Zeiss inverted microscope (Carl Zeiss Microscopy, LLC, Thornwood, NY, USA). Images were collected every 20 to 30 min, and select images are shown (Figs. 3 and 4). A 405 nm diode laser and a 488 nm argon laser were used for excitation. Fluorescence emission was collected with a 10× or 40× objective and was represented using pseudo colors for three channels: 410 to 469 nm (blue), 504 to 581 nm (green), and 592 to 759 nm (red). The resulting images were analyzed using the Zen software (Carl Zeiss Microscopy) to measure the changes of cell wall thickness.

### Statistical analysis

Statistical analyses were calculated using ProStat (v 5.01, Poly Software International, Pearl River NY, USA). Significance is indicated with the following: *P* < 0.05*, *P* < 0.01**, *P* < 0.005***, *P* < 0.001****. Multiple comparisons were done with One-way ANOVA with post-hoc Tukey’s HSD. Full results of the statistical analysis can be found in the table and figure legends.

## Additional files

Additional file 1:
**Movie 1.** Confocal fluorescence imaging of *WT Arabidopsis* during [C_2_C_1_im][OAc] pretreatment at 140 °C. Autofluorescence of 100 μm slices of the stems from during [C_2_mim][OAc] pretreatment at 140 °C over 3 hours. The temperature ramp from ambient conditions to 140 ± 5 °C occurring during time 0 to 46 min. 5× magnification, 10 frames per second. Additional file 2:
**Movie 2.** Confocal fluorescence imaging of *LLL Arabidopsis* during [C_2_C_1_im][OAc] pretreatment at 140 °C. Autofluorescence of 100 μm slices of the stems from during [C_2_mim][OAc] pretreatment at 140 °C over 3 hours. The temperature ramp from ambient conditions to 140 ± 5 °C occurring during time 0 to 46 min. 5× magnification, 10 frames per second. Additional file 3:
**Movie 3.** Confocal fluorescence imaging of *LLHPL1 Arabidopsis* during [C_2_C_1_im][OAc] pretreatment at 140 °C. Autofluorescence of 100 μm slices of the stems from during [C_2_mim][OAc] pretreatment at 140 °C over 3 hours. The temperature ramp from ambient conditions to 140 ± 5 °C occurring during time 0 to 46 min. 5× magnification, 10 frames per second. Additional file 4:
**Movie 4.** Confocal fluorescence imaging of *LLHPL2 Arabidopsis* during [C_2_C_1_im][OAc] pretreatment at 140 °C. Autofluorescence of 100 μm slices of the stems from during [C_2_mim][OAc] pretreatment at 140 °C over 3 hours. The temperature ramp from ambient conditions to 140 ± 5 °C occurring during time 0 to 46 min. 5× magnification, 10 frames per second. Additional file 5: Figure S1. Enlarged view of [C_2_C_1_im][OAc] pretreatment on *Arabidopsis* (a, *WT*, b, *LLL*, c, *LLHPL1*, d, *LLHPL2*) for pretreatment at 140 °C at 3 hours with temperature increase from ambient to 140 ± 5 °C during the first 30 min, scale bar 500 μm. Additional file 6: Figure S2. Analysis of confocal imaging of autofluorescence comparing cell wall swelling during IL pretreatment with [C_2_C_1_im][OAc] at 70 °C for 12 hours of 100 μm slices from *Arabidopsis* engineered lines as measured by change of area min normalized to 0 and max normalized to 1. Data shown for 3 individual swelling experiments (two separate experiments on the *LLHPL2* engineered line (LLHPL2a and LLHPL2b and) and one *WT*). Area was calculated in (ImageJ, NIH). The changing in swelling ends around 5 hours and remains relatively constant for the next 6 hours for all of the lines. 5 hours was chosen as the duration for the pretreatment at 70 °C. Additional file 7:
**Movie 5.** Confocal fluorescence imaging of *WT Arabidopsis* during [C_2_C_1_im][OAc] pretreatment at 70 °C. Autofluorescence of 100 μm slices of the stems from during [C_2_mim][OAc] pretreatment at 70 °C over 24 hours. The temperature ramp from ambient conditions to 70 ± 5 °C occurring during time 0 to 46 min. 5× magnification, 10 frames per second. Additional file 8:
**Movie 6.** Confocal fluorescence imaging of *LLHPL2 Arabidopsis* during [C_2_C_1_im][OAc] pretreatment at 70 °C. Autofluorescence of 100 μm slices of the stems from during [C_2_mim][OAc] pretreatment at 70 °C over 24 hours. The temperature ramp from ambient conditions to 70 ± 5 °C occurring during time 0 to 46 min. 5× magnification, 10 frames per second. Additional file 9: Tables S1 and S2. Table of Tukey’s HSD post-hoc comparison of glucose, xylose or total sugar recovered between the different engineered lines of *Arabidopsis* versus each other at each pretreatment condition. Table of Tukey’s HSD post-hoc comparison of glucose, xylose or total sugar recovered versus pretreatment condition versus the different engineered lines of *Arabidopsis*.

## Abbreviations

ASR: acid soluble residue
IL: ionic liquid
LLL: low lignin line
LLHPL1: low lignin high polysaccharide lines
WT: wild type *Arabidopsis*
[C_2_C_1_im][OAc]: 1-ethyl-3-methylimidazolium acetate
