# Supplementary figures and images for: Restricting lignin and enhancing sugar deposition in secondary cell walls enhances monomeric sugar release after low temperature ionic liquid pretreatment
Source: Biotechnol Biofuels. 2015 Jul 4;8:95. doi: 10.1186/s13068-015-0275-2 (PMC4496950; doi:10.1186/s13068-015-0275-2)

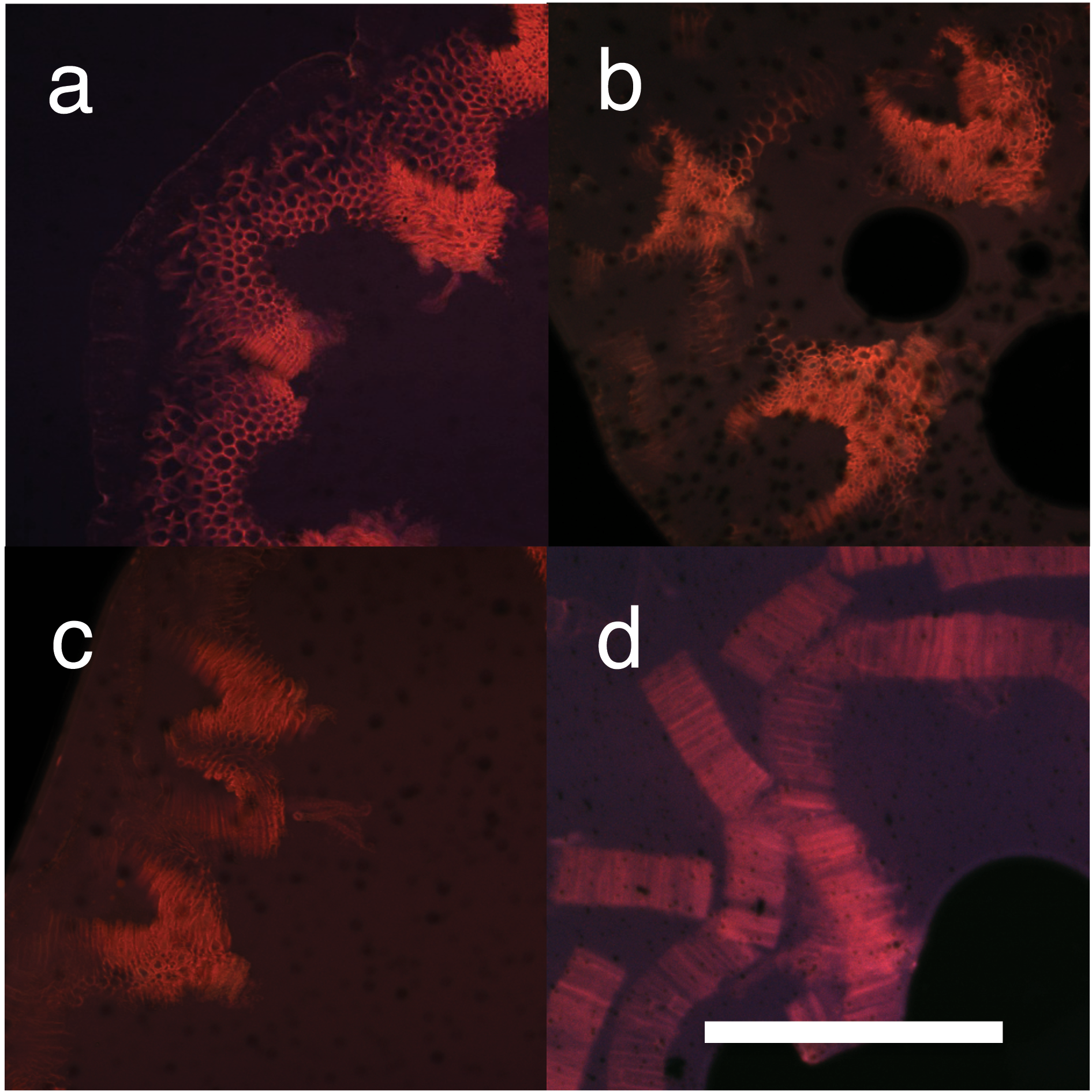

Supplement: Additional file 5: Figure S1. — Enlarged view of [C2C1im][OAc] pretreatment on Arabidopsis (a, WT, b, LLL, c, LLHPL1, d, LLHPL2) for pretreatment at 140 °C at 3 hours with temperature increase from ambient to 140 ± 5 °C during the first 30 min, scale bar 500 μm. [file 13068_2015_275_MOESM5_ESM.png]

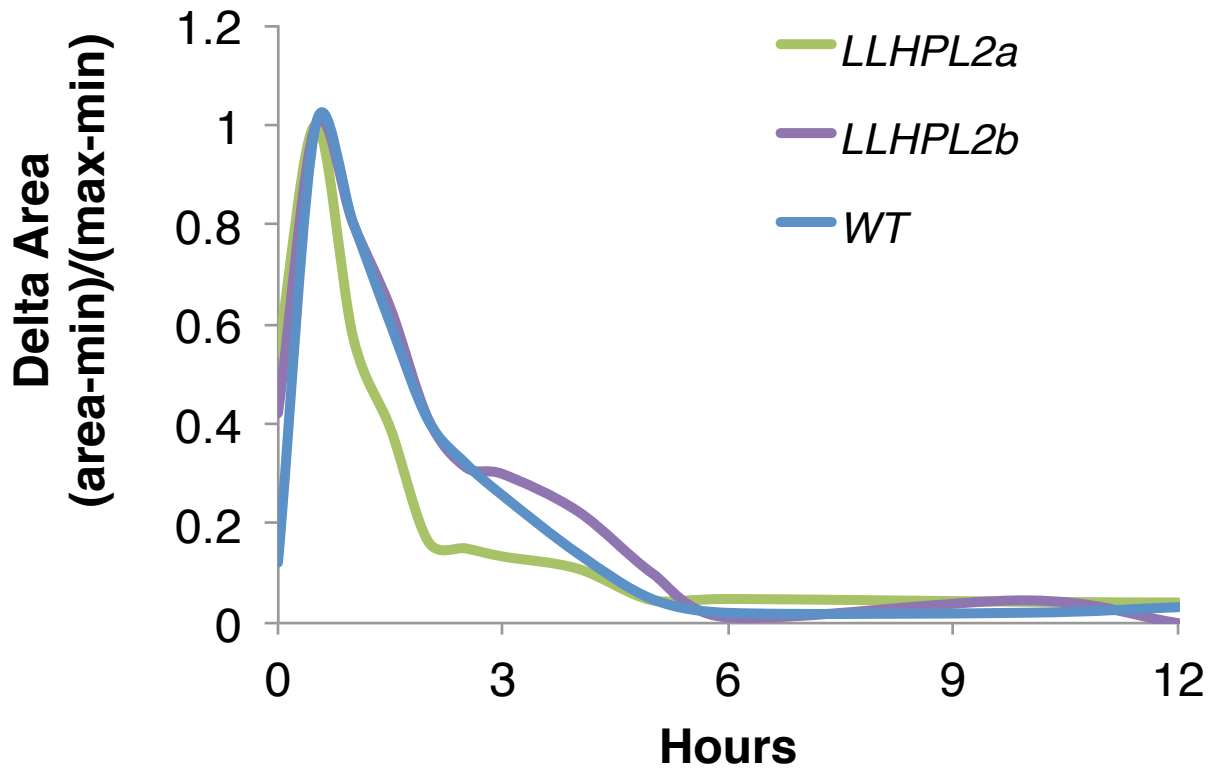

Supplement: Additional file 6: Figure S2. — Analysis of confocal imaging of autofluorescence comparing cell wall swelling during IL pretreatment with [C2C1im][OAc] at 70 °C for 12 hours of 100 μm slices from Arabidopsis engineered lines as measured by change of area min normalized to 0 and max normalized to 1. Data shown for 3 individual swelling experiments (two separate experiments on the LLHPL2 engineered line (LLHPL2a and LLHPL2b and) and one WT). Area was calculated in (ImageJ, NIH). The changing in swelling ends around 5 hours and remains relatively constant for the next 6 hours for all of the lines. 5 hours was chosen as the duration for the pretreatment at 70 °C. [file 13068_2015_275_MOESM6_ESM.pdf]
