# Supplementary material for: Restricting lignin and enhancing sugar deposition in secondary cell walls enhances monomeric sugar release after low temperature ionic liquid pretreatment
Source: Biotechnol Biofuels. 2015 Jul 4;8:95. doi: 10.1186/s13068-015-0275-2 (PMC4496950; doi:10.1186/s13068-015-0275-2)
Supplement: Additional file 9: Tables S1 and S2. — Table of Tukey’s HSD post-hoc comparison of glucose, xylose or total sugar recovered between the different engineered lines of Arabidopsis versus each other at each pretreatment condition. Table of Tukey’s HSD post-hoc comparison of glucose, xylose or total sugar recovered versus pretreatment condition versus the different engineered lines of Arabidopsis. [file 13068_2015_275_MOESM9_ESM.docx]

**Supplementary Table 1**: Table of Tukey’s HSD post-hoc comparison of glucose, xylose or total sugar recovered between the different engineered lines of *Arabidopsis* versus each other at each pretreatment condition.

|  | Glucose | | | Xylose | | | Total | | |
| --- | --- | --- | --- | --- | --- | --- | --- | --- | --- |
|  | ut | 70 °C | 140 °C | ut | 70 °C | 140 °C | ut | 70 °C | 140 °C |
| *WT* vs *LLL* | p<0.05 | ns | p<0.05 | p<0.01 | p<0.01 | ns | p<0.01 | p<0.05 | p<0.05 |
| *WT* vs *LLHPL1* | p<0.01 | p<0.01 | ns | p<0.01 | p<0.01 | ns | p<0.01 | p<0.01 | ns |
| *WT* vs *LLHPL2* | p<0.01 | ns | p<0.05 | p<0.01 | p<0.01 | ns | p<0.01 | p<0.01 | ns |
| *LLL* vs *LLHPL1* | p<0.01 | p<0.05 | ns | p<0.01 | p<0.01 | ns | p<0.01 | ns | ns |
| *LLL* vs *LLHPL2* | ns | ns | ns | ns | ns | ns | ns | ns | ns |
| *LLHPL1* vs *LLHPL2* | p<0.01 | p<0.05 | ns | p<0.01 | p<0.01 | ns | p<0.01 | ns | ns |

**Supplementary Table 2:** Table of Tukey’s HSD post-hoc comparison of of glucose, xylose or total sugar recovered versus pretreatment condition versus the different engineered lines of *Arabidopsis*.

|  | *WT* | *LLL* | *LLHPL1* | *LLHPL2* |
| --- | --- | --- | --- | --- |
| glucose | | | | |
| Ut vs 70 | p<0.01 | p<0.01 | p<0.01 | p<0.01 |
| Ut vs 140 | p<0.01 | p<0.01 | p<0.01 | p<0.01 |
| 70 vs 140 | p<0.05 | ns | ns | ns |
| xylose | | | | |
| Ut vs 70 | p<0.01 | p<0.01 | p<0.01 | p<0.01 |
| Ut vs 140 | p<0.01 | ns | ns | p<0.01 |
| 70 vs 140 | p<0.01 | p<0.05 | p<0.01 | p<0.01 |
| total | | | | |
| Ut vs 70 | p<0.01 | p<0.01 | p<0.01 | p<0.01 |
| Ut vs 140 | p<0.01 | p<0.01 | ns | ns |
| 70 vs 140 | p<0.01 | ns | p<0.01 | p<0.01 |
